# Age-related association of venom gene expression and diet of predatory gastropods

**DOI:** 10.1186/s12862-016-0592-5

**Published:** 2016-01-28

**Authors:** Dan Chang, Thomas F. Duda

**Affiliations:** Department of Ecology and Evolutionary Biology and Museum of Zoology, University of Michigan, Ann Arbor, Michigan USA; Department of Statistics, University of Michigan, Ann Arbor, Michigan USA; Smithsonian Tropical Research Institute, Balboa, Ancόn Republic of Panama; Present address: University of California Santa Cruz, 1156 High Street -- Mail Stop EEBiology, Santa Cruz, CA 95064 USA

**Keywords:** *Conus*, Conotoxin, Developmental plasticity, Predator–prey interactions

## Abstract

**Background:**

Venomous organisms serve as wonderful systems to study the evolution and expression of genes that are directly associated with prey capture. To evaluate the relationship between venom gene expression and prey utilization, we examined these features among individuals of different ages of the venomous, worm-eating marine snail *Conus ebraeus*. We determined expression levels of six genes that encode venom components, used a DNA-based approach to evaluate the identity of prey items, and compared patterns of venom gene expression and dietary specialization.

**Results:**

*C. ebraeus* exhibits two major shifts in diet with age—an initial transition from a relatively broad dietary breadth to a narrower one and then a return to a broader diet. Venom gene expression patterns also change with growth. All six venom genes are up-regulated in small individuals, down-regulated in medium-sized individuals, and then either up-regulated or continued to be down-regulated in members of the largest size class. Venom gene expression is not significantly different among individuals consuming different types of prey, but instead is coupled and slightly delayed with shifts in prey diversity.

**Conclusion:**

These results imply that changes in gene expression contribute to intraspecific variation of venom composition and that gene expression patterns respond to changes in the diversity of food resources during different growth stages.

**Electronic supplementary material:**

The online version of this article (doi:10.1186/s12862-016-0592-5) contains supplementary material, which is available to authorized users.

## Background

Phenotypes for resource acquisition may evolve in response to changes in resource availability or utility. Gill rakers of alewives [1], drilling behavior of marine snails [2], venoms of snakes [3–5] and beaks of Darwin’s finches [6] all exhibit specific phenotypes that correspond to particular resources. But the genetic mechanisms underlying these phenotypic changes are mostly unknown. In addition to non-synonymous mutations, gene regulation also influences phenotypic changes. Expression of genes that contribute to the ability to consume particular resources is often regulated by characteristics of the resources [7, 8]. For example, for groups of mice that are fed with the same food items as those consumed by human and chimp, levels of differential expression in liver tissues of these mice are comparable to levels of differential expression between liver tissues of human and chimp [9]. This indicates that dietary changes exert more influence on gene expression than the inherent regulating mechanisms among species. To understand the genetic mechanisms underlying the dynamics of predator–prey interactions, it is essential to evaluate the effect of diets on expression of genes that are directly involved in resource utilization.

The developmental process, accompanied by drastic changes of phenotypes with age, represents an ideal case to explore the connection between gene expression and environmental cues [10]. Predatory marine snails of the family Conidae (‘cone snails’) exhibit particular dietary changes that are associated with increase in body size [11, 12], and many venom genes used for predation have already been well characterized [13, 14]. Leviten [11] suggested that diets of vermivorous *Conus* species shift from being trophic specialists as juveniles, to generalists as subadults, and then to specialists as adults. Characters associated with predation also exhibit vast changes during development. For example, radular teeth of *Conus magus* that are used to inject venom into prey, are morphologically distinct in juvenile and adult stages that specialize on polychaetes and fish respectively [15–17]. Cone snails use venom, a cocktail of numerous compounds including conotoxins, to capture prey and, for some species, to defend against predators [13, 18]. Conotoxins genes undergo extensive gene duplication and rapid evolution [19, 20], and their expression patterns are highly divergent among species [21–23]. Similar to the changes of radula teeth through development, the quantity and diversity of conotoxins may change with growth, but no prior study has tested this hypothesis.

To investigate patterns of changes of conotoxin gene expression and diet among individuals of different shell sizes, we chose *Conus ebraeus*, a vermivorous species, as our study organism. This species is abundant at numerous shallow water sites in the Indo-West Pacific and its diet has been studied previously [11, 12, 24]. We also have sequence alignments of several conotoxin genes from previous population genetic and molecular evolution studies of this species [22, 24–27], all of which facilitate the experimental design of this study.

We specifically addressed the following questions. Do conotoxin gene expression patterns differ among individuals of different ages? If so, how does expression change through time? Are some genes uniquely expressed only in particular stages? Do shifts in conotoxin gene expression patterns correspond with shifts in diet? If so, are the dietary shifts associated with changes in the types of prey or diversities of prey items? To answer these questions, we collected individuals from a single population at Guam, determined the identity of prey species based on microscopic examination of feces and a DNA-based approach, quantified conotoxin gene expression levels among individuals of different shell sizes, and evaluated the relationship between shifts in conotoxin gene expression and diet.

## Methods

### Specimens

We collected specimens of *C. ebraeus* at Pago Bay, Guam in May 2010. We measured shell lengths of each specimen upon collection. We placed individual specimens in separate cups that contained enough seawater to cover the animal, collected feces upon defecation, and preserved feces in the 95 % ethanol. Members of this species typically consume only one prey item every other night [28], and therefore each of our fecal samples usually contains the remains of a single prey individual [24]. After collecting feces we returned most samples back to their original collecting location at Pago Bay, and dissected 60 samples following the permit of Guam Department of Agriculture. We determined sexual maturity and sex of each specimen based on the presence/absence of a penis. We preserved venom ducts in RNAlater (Ambion, Inc.) and stored them at −20 °C prior to preparation of cDNA.

### Identification of prey items

**Fig. 1:**
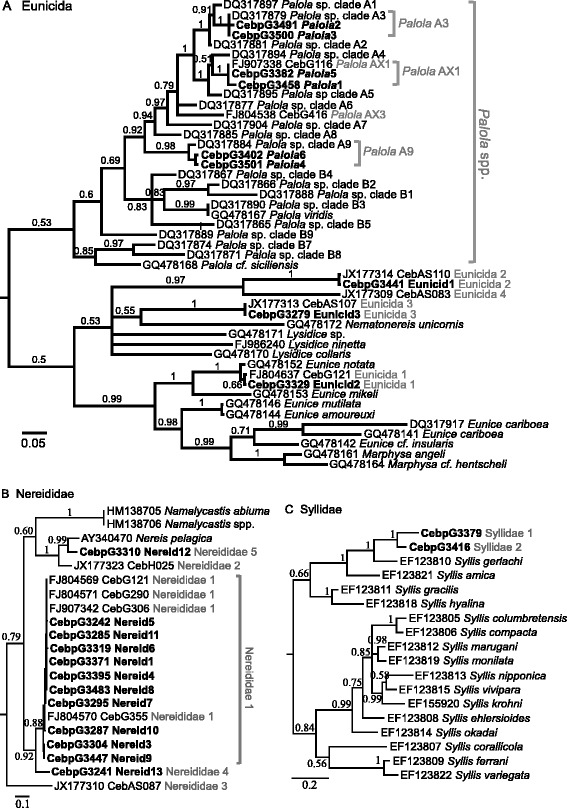
Phylogenies of 16S rRNA sequences of fecal samples of *C. ebraeus* and known polychaete species. Bayesian posterior probabilities are labeled at nodes of major clades. Sequences downloaded from GenBank are labeled with their respective accession numbers. Sequences obtained in this study are highlighted in bold. Putative prey species are labeled in grey next to the sequence names. **a** Phylogeny of species of the order Eunicida with GTR [76] + I + G model. **b** Phylogeny of species of the family Nereididae with GTR + G model. **c** Phylogeny of species of the family Syllidae with GTR + G model

We examined feces from 243 individuals with microscopy to determine tentative identifications of prey items. Then we used a DNA-based approach described by Duda et al. [24] to further evaluate identifications. In brief, we obtained sequences of a region of the mitochondrial 16S ribosomal RNA gene from DNA extracts of feces, and aligned these with 16S rRNA sequences of polychaetes downloaded from GenBank (accession numbers shown in Fig. 1) in Se-Al 2.0 [29]. We obtained the relative positions of fecal sequences in neighbor-joining trees and from these results assigned fecal sequences to major taxonomic groups of Polychaeta (e.g., Eunicida, Nereididae and Syllidae). We selected the best substitution models with the Bayesian Information Criterion in jModelTest v0.1.1 [30] for alignments of 16S gene sequences for each of the taxonomic groups, and built Bayesian consensus phylogenies for each group separately with these models (10,000,000 generations, two runs, four chains, 25 % burn-in) in MrBayes v3.1.2 [31]. We ultimately determined the identity of prey species based on the sequence similarity and phylogenetic positions of fecal sequences with sequences of known or pre-defined polychaete species in these estimated species phylogenies.

### Analysis of dietary data

Shell lengths provide an approximate estimate of ages of *Conus* individuals [11, 12]. To determine if individuals of different sizes show differences in prey selection, we performed one-way Analysis of Variance (ANOVA) of shell lengths of individuals consuming different prey species and higher taxonomic levels with the function *lm* in R v2.15.0 [32]. To identify patterns of transition in dietary composition, we built a heatmap of percentages of each prey species captured by individuals of a specific shell length bin with the *heatmap.2* function in the *gplots* package [33]. We used Shannon-Weiner (*H’*) [34] and Gini-Simpson’s (*S*) [35] indices and average genetic distances (GD) to quantify levels of prey diversity in each size bin. The two parameters *H’* and *S* were estimated with the function *diversity* in the package *vegan* [36]. To calculate GD, we estimated pairwise genetic distances of the mitochondrial 16S rRNA sequences of prey species with the Tamura-Nei [37] + G distance model and complete deletion of gaps in MEGA 5.05 [38], and computed the average genetic distances. We performed sliding-window analyses of *H’*, *S* and GD of dietary compositions with a window size of 5 mm in shell lengths.

We used *F*-statistics to evaluate patterns of genetic differentiation of dietary compositions among groups of individuals representing different size classes. We constructed these groups with a sliding window approach with a window size of 5 mm in shell lengths. We estimated pairwise *Φ*_ST_ values (‘*D*_ST_’ values) as measures of the phylogenetic disparity of prey, of 16S rRNA sequences of prey items within each group/window in Arlequin 3.1 [39] with the Tamura-Nei distance model [37]. *P*-values were estimated from Monte Carlo simulations of 10,100 replicates. We built a heatmap of absolute *D*_ST_ values along the gradient of shell lengths with the approach described above.

We defined ranges of shell lengths of small, medium and large individuals that correspond with inferred dietary transitions. This was achieved by defining the inflection points of increasing (or decreasing) trends in sliding window analyses of dietary diversities as well as the significance of the extent of genetic differentiation (as measured by *D*_ST_ values) as boundaries of different size classes. We tested if the three size classes show differences in dietary composition by performing Fisher’s exact tests [40] with the *fisher.test* function. *P*-values were determined from results of 100,000 simulated datasets under the null hypothesis of no difference between groups.

### Quantification of conotoxin gene expression

We extracted mRNA from venom ducts of 60 *C. ebraeus* individuals (with shell lengths ranging from 7 to 26 mm) and prepared complementary DNA (cDNA) following the approach described in Duda and Palumbi [19]. We examined six conotoxin genes that were identified from population studies of this species in the Indo-West Pacific [24, 27]: locus E1 (an O-superfamily locus that putatively encodes an ω-conotoxin), locus EA1 and EA4 (two A-superfamily loci that putatively encode α-conotoxins), locus ED4, ED8 and ED20 (three O-superfamily loci that putatively encode δ-conotoxins) (GenBank accession numbers JX177193, JX177106, JX177246, JX177272, JX177276, JX177278). These genes are expected to be single-copy, conform to Hardy-Weinberg Equilibrium among populations; alleles of these genes of the Guam population have already been well characterized [27].

We used quantitative PCR (qPCR) to measure expression levels of the six conotoxin genes. The ‘carryover’ or contamination of genomic DNA in prepared cDNA can inflate expression levels of genes measured by qPCR. To avoid the interference of this ‘genomic DNA carryover’ in the quantification of gene expression, we specifically designed sets of primers that span known intron positions that should prevent any amplification from gDNA templates. We designed locus-specific reverse primers annealing to the toxin-coding region downstream to intron(s), and paired them with general forward primers that anneal within conserved regions upstream of the intron position(s) (i.e., within the signal or prepro region of the genes) (Additional file 1: Table S1). We tested specificity of these primer sets by amplifying and directly sequencing individuals with known genotypes.

We chose a β-tubulin gene as the endogenous control and estimated abundance of its gene transcripts with qPCR and primers specific for this locus (forward primer 5′ACAGCAGCTACTTTGTTGAATGGAT3′ and reverse primer 5′CAGTGTACCAATGGAGGAAAGCC3′). Expression levels of the β-tubulin gene, a ‘house-keeping’ gene, should be stable regardless of cells, tissue or developmental stages [41]. We added Tris-EDTA buffer to each cDNA preparation to a total volume of 175 μL and aliquoted an equal volume of cDNA samples for each qPCR run. We used SYBR Green chemistry to detect and quantify amplified products. All qPCR runs were performed on an ABI Prism 7500 machine at the University of Michigan School of Dentistry’s Molecular Biology Core Laboratory. To reduce the effect of noise associated with measurements, each round of qPCR was performed on three replicates for each individual; we used average results of the three replicates as estimates of expression levels. The amplification procedure included ten minutes of initial denaturing at 95 °C, followed by 40 cycles of amplification: denaturing at 95 °C for 15 s, annealing at 54 °C or 60 °C for 30 s, and elongation at 72 °C for 35 s in which fluorescent signals were collected. The annealing temperature of the β-tubulin locus and four conotoxin genes was 54 °C; for conotoxin loci E1 and ED4, the annealing temperature was 60 °C. We added a dissociation stage (95 °C for 15 s, 60 °C for 1 min, and 95 °C for 15 s for each sample) at the end of each run to evaluate the specificity of amplifications. The dissociation stage measures temperatures at which amplified products re-nature. Multiple temperatures of renaturation imply the presence of multiple unique DNA fragments and non-specific amplification. To ensure similarity in efficiency of primers of conotoxin genes with primers of the β-tubulin gene, we made 1/5 and 1/25 dilutions of cDNA samples of 12 individuals, and compared efficiencies of these primers following the approach described by Schmittgen and Livak [42].

We used the comparative C_T_ method [42] to estimate expression levels of the six conotoxin genes relative to the endogenous house-keeping β-tubulin gene. C_T_ values of some samples were labeled as ‘undetermined’ (the amplification did not reach the threshold by the last PCR cycle), and only very small amounts of target cDNA were amplified. To simplify the calculation, we converted ‘undetermined’ results to 40 (i.e., the last PCR cycle). We estimated ∆C_T_ values of each conotoxin gene relative to the endogenous gene of each individual by subtracting average C_T_ values of conotoxin genes among three replicates with that of the β-tubulin gene, and calculated relative expression levels of conotoxin genes with the formula ${2}^{-\varDelta {\mathrm{C}}_{\mathrm{T}}}$.

### Analysis of patterns of conotoxin gene expression

To normalize the conotoxin gene expression levels for statistical analyses, we used -∆C_T_ as an approximation to the log transformation of expression levels of conotoxin genes relative to those of the β-tubulin gene $\left( \log\;\left({2}^{-\varDelta {\mathrm{C}}_{\mathrm{T}}\ }\right)\right)$. We constructed a heatmap with *C. ebraeus* individuals as row variables, conotoxin loci as column variables, and absolute and scaled values of -∆C_T_ as input. For samples that exhibited no amplification of the β-tubulin gene or conotoxin genes, we considered them to represent low quality cDNA preparations and eliminated them from subsequent analyses. We measured Euclidean distances of relative expression levels of conotoxin genes among samples with the function *daisy* in the package *cluster* [43]. Then we performed hierarchical clustering analyses with Wald’s method [44] with the function *agnes* in the package *cluster* to identify potential hierarchical structures of conotoxin gene expression.

### Analysis of the association of shifts of diets and venom

We used boxplots of expression levels (−∆C_T_ values) and one-way ANOVA to test if expression levels differ among samples consuming different prey species or higher taxonomic categories. We performed hierarchical clustering analysis with Wald’s method on this ‘reduced’ dataset, and tested if dietary composition differs between clusters with Fisher’s Exact Tests as described above.

To compare patterns of ontogenetic change of conotoxin gene expression and dietary composition, we centered and standardized results of sliding window analyses (the windows size of 5 mm) of -∆C_T_ values of all conotoxin genes as well as dietary diversity estimators *H’*, *S* and *GD*. We viewed the increase of shell lengths as the progress of time/ages, and treated results of sliding-window analyses of expression levels of each conotoxin gene and estimates of dietary diversity of individuals of different shell lengths as time series. We tested if each time series of conotoxin gene expression is positively correlated with, plus a possible lead or lag of, the time series of dietary diversities using the cross-correlation *ccf* function, and verified the significance of results with a linear regression model (function *lm*) in R. The R scripts used in this study are available (from DC) upon request.

## Results

### Identification of prey species

Out of the possible fecal materials collected from 243 individuals of *C. ebraeus*, samples from 151 individuals contained hard parts of polychaetes that were identifiable from microscopic examination. Based on the morphological characteristics of these hard parts, we determined 86 samples to be of the family Nereididae, six of the family Syllidae, one of the family Terebellidae, and 49 of the order Eunicida, including 27 of the genus *Palola*. We obtained 16S rRNA sequences from DNA extracts of 54 fecal samples (Additional file 1: Table S2). Based on the similarities of these sequences with sequences of known annelid species and species defined in previous studies of diets of *C. ebraeus* [27, 45], we determined that the 54 fecal samples represent six Eunicida species (three *Palola* species), three Nereididae species and two Syllidae species (Fig. 1). Two species inferred from the phylogeny, ‘*Palola* A3’ and ‘*Palola* A9’, were previously characterized by Schulze [46]. Five inferred polychaete species, ‘*Palola* AX1’, ‘Eunicida 1’, ‘Eunicida 2’, ‘Eunicida 3’ and ‘Nereididae 1’, were found in dietary studies of *C. ebraeus* adults at Guam and American Samoa [27].

### Age-related shift of diet

**Fig. 2:**
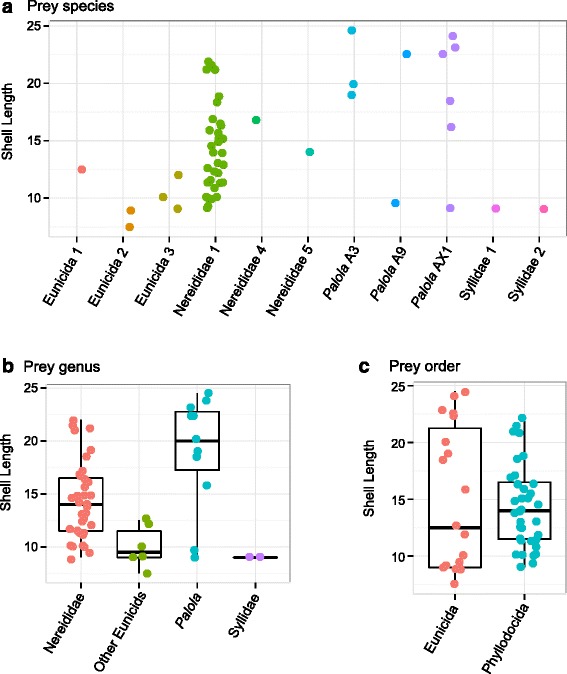
Scatterplots and superimposed boxplots of shell lengths (mm) of *C. ebraeus* individuals consuming different types of prey. **a** Prey species, **b** prey genera, **c** prey orders

Nereididae and *Palola* species are consumed by a wide size range of *C. ebraeus* individuals (Fig. 2). The relatively rare species (‘Eunicida 1’, ‘Eunicida 2’, ‘Eunicida 3’, ‘Syllidae 1’ and ‘Syllidae 2’) are mostly consumed by small individuals. Different species and inferred genera of prey are targeted by predators of significantly different shell sizes (*P*-value = 0.011 for groups divided by prey species, with an average shell length range of 8-21 mm; *P*-value < 0.0001 for groups divided at prey genera, with an average shell length range of 9-19 mm; Fig. 2a-b). The average difference in shell lengths among prey types disappears when evaluated at the order level (Fig. 2c).

**Fig. 3:**
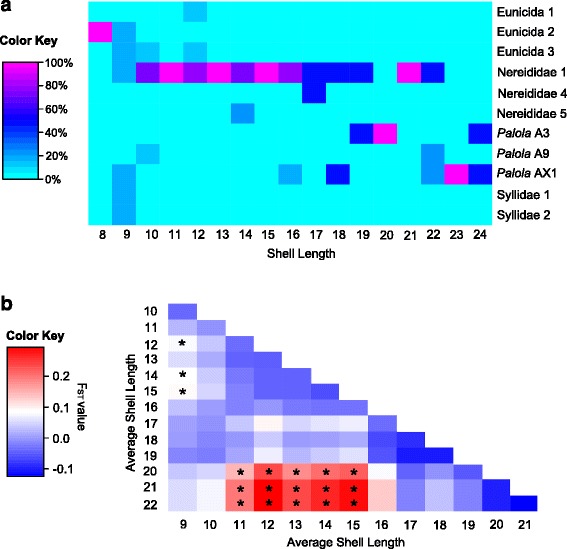
Heatmaps of dietary ontogeny of *C. ebraeus* individuals. **a** Heat map of frequencies of prey species consumed by *C. ebraeus* individuals of the same shell lengths. **b** Heat map of pairwise *D*_ST_ values between size classes of sliding-window analyses (window size = 5 mm). *P*-values are estimated with simulations of 10,100 replicates, and significant results (*P*-value < 0.05) are labeled with asterisks in the cells

The diversity of prey differs among different size classes. Based on the inflection points of sliding window results of dietary diversity (Fig. 5a), we defined individuals with shell lengths smaller than 11 mm as ‘small’, those with shell lengths between 11 and 17 mm as ‘medium-sized’, and those larger than 17 mm in shell lengths as ‘large’. Small individuals exhibit the broadest dietary spectrum, medium-sized ones specialize mostly on Nereididae species, and large ones prey on both Nereididae and *Palola* species (Fig. 3a). Medium-sized individuals possess significantly different dietary composition in comparison to individuals of other size ranges, as illustrated by the sliding window analysis of phylogenetic disparity values *D*_ST_ (Fig. 3b) and Fisher’s exact tests of prey species among the three size classes (Additional file 1: Table S3).

### Age-related shift of conotoxin gene expression

**Fig. 4:**
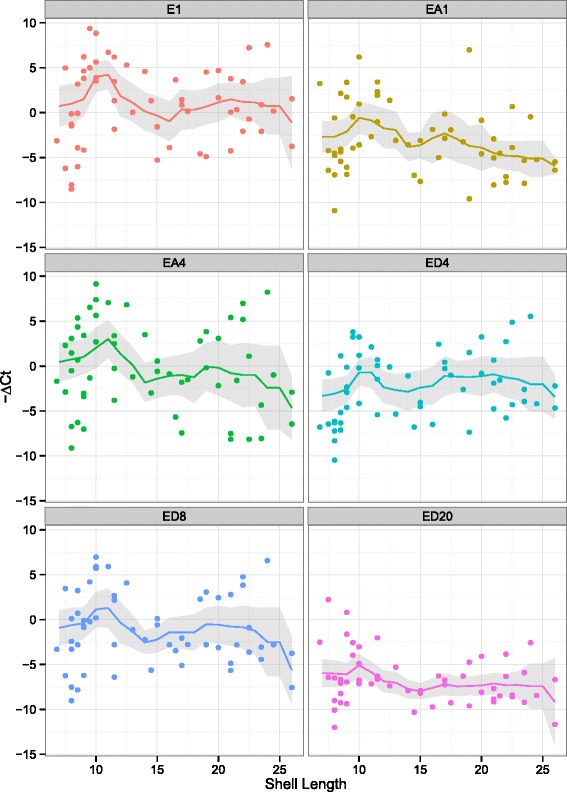
Relative levels of expression of the six conotoxin genes against shell lengths. The trend lines represent average values calculated from sliding window analyses (window size = 5 mm), and the shades are confidence intervals of the mean

**Fig. 5:**
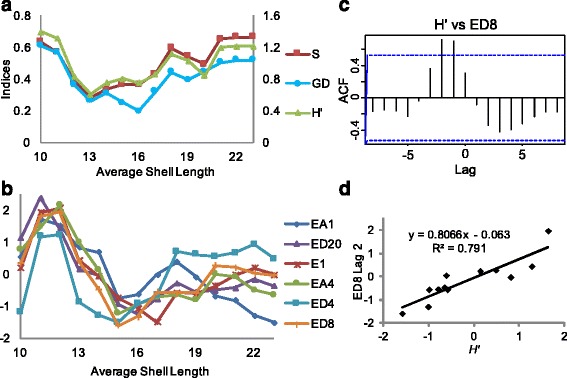
Patterns of ontogenetic shifts of dietary diversities and levels of conotoxin gene expression. Average levels of expression of six conotoxin genes and dietary diversities are calculated with a sliding window approach (with window size of 5 mm in shell lengths). **a** Plot of dietary variables versus average shell lengths. Shannon’s index (*H’*), Gini-Simpson’s index (*S*), and average genetic distances (GD). The Y-axis on the left represents *S* and GD, whereas the Y-axis on the right represents *H’*. **b** Plot of relative levels of expression of six conotoxin loci EA1, ED20, E1, EA4, ED4, and ED8 versus shell lengths. The expression levels are centered and standardized. **c** Cross-correlation of conotoxin gene expression levels and dietary diversities through increasing shell lengths, using conotoxin locus ED8 and dietary variable *H’* as an example. Cross-correlations of all conotoxin genes and dietary variables are illustrated in Additional file 1: Figure S5. Y-axis: correlation coefficient of two series; X-axis: lag in shell lengths of *H’* in comparison to conotoxin gene expression; blue dashed lines: 95 % confidence intervals. **d** Linear regression of lag of expression levels of locus ED8 with the dietary variable *H’* by a time period equivalent to 2 mm in shell lengths. Regression analyses of expression levels of all conotoxin genes and dietary variables are shown in Additional file 1: Figure S6

We eliminated three individuals from analyses of conotoxin gene expression because these individuals yielded poor quality cDNA or included some failed reactions (Additional file 2). The remaining 57 individuals exhibit considerable variation in expression for the six conotoxin genes evaluated (Figs. 4 and 5). Expression levels are highest for small individuals and lowest in medium-sized ones (Figs. 4 and 5b). Hierarchical clustering analyses divided individuals into two major groups (Additional file 1: Figure S1). Cluster 1 contains individuals of a relatively even size distribution, whereas cluster 2 is composed exclusively of individuals at the two extremes of the size distribution (i.e., small and large individuals). Members of cluster 2 primarily exhibited higher levels of conotoxin gene expression than those of the cluster 1.

Sliding window analyses revealed that average expression levels of conotoxin genes initially decrease and then increase with sizes of individuals (Figs. 4 and 5b). Expression levels of four genes ED4, ED8, E1 and EA4 are highest in small individuals and lowest in medium-sized individuals; expression levels increase in large individuals but do not reach the same levels as in small individuals (Fig. 5b). Average expression levels of loci EA1 and ED20 also decrease in medium-sized individuals, but do not show the same increasing pattern in large individuals as exhibited by the other genes (Fig. 5b).

### Association between conotoxin gene expression and diet

Examination of conotoxin gene expression of 35 individuals whose prey items were also determined revealed no association between conotoxin gene expression levels and specific prey species. Expression levels of most conotoxin genes did not differ significantly among individuals consuming different prey taxa; the only exception is locus ED20 with a *P*-value = 0.006 (Additional file 1: Figure S2). No significant differences in conotoxin gene expression levels (including locus ED20) were detected among groups of individuals determined based on higher taxonomic levels of their prey (Additional file 1: Figures S3 and S4). The hierarchical clustering approach divided these individuals into two major clusters that exhibit no significant differences in prey utilization (*P*-value of the Fisher’s exact test is 0.270). Examination of the samples of the medium-sized and large groups, which only consumed Nereididae and *Palola* species, did not reveal any significant association between levels of conotoxin gene expression and dietary specialization (data not shown).

Patterns of variation in conotoxin gene expression and dietary diversity are similar in that both exhibit a trend of decrease and then increase with increases in shell lengths (Fig. 5a-b). However, the inflection points of the two time series are not coincident. Dietary changes lead changes in conotoxin gene expression by the amount of time equivalent to the growth time of one or two millimeters in shell length (Fig. 5c; Additional file 1: Figure S5). This pattern is confirmed by the significantly positive coefficients in cross-correlation and linear regression, tests applied to determine if dietary diversity of individuals is correlated with conotoxin gene expression levels of individuals one or two mm larger (Fig. 5c-d, Additional file 1: Figures S5 and S6). The only exception is conotoxin gene EA1; although changes in expression of this gene seem to lag changes in dietary diversity, no significant correlation was detected between the two variables (Additional file 1: Figure S5B).

## Discussion

Through examination of fecal samples of over 200 *C. ebraeus* individuals and quantification of mRNA abundance of six conotoxin genes of 58 individuals of different sizes, we reconstructed time series of changes in dietary diversity and venom gene expression of *C. ebraeus* at Guam. The diversity of prey species targeted by these snails changes from high to low and then to high again with growth, while expression levels of conotoxin genes appear to be up-regulated in small individuals, down-regulated in medium-sized ones, and either go up or stay down in large individuals. We detected significant association between time series of conotoxin gene expression levels and sequential changes of dietary diversity, but the inflection points in conotoxin gene expression are delayed relative to dietary changes.

The observed patterns of changes in prey utilization with growth may be affected by three factors: competition and associated microhabitat differentiation, different body volume and energy efficiency of prey types, or minimization of predation risk. Intraspecific competition among congeners of different sizes, as well as interspecific competition at a particular growth stage, often reduce niche overlap and promote resource partitioning within an age-structured population [11, 47–50]. In addition, body size of predators determines the size of prey that is consumed [47, 48, 51, 52]. Cone snails engulf prey entirely, and therefore consuming prey that are larger than their handling capacity is costly [11]. Among prey items of *C. ebraeus* at Guam, members of the family Eunicidae typically exhibit the largest body sizes, followed by Nereididae and Syllidae [53], and these families of polychaetes are coincidently targeted by large, medium and small-sized cone snails as observed here. Moreover, prey handling time is usually shorter than searching time for many *Conus* species [11], and therefore small *C. ebraeus* may expand their dietary spectra to minimize their exposure to predation while searching for food.

Intrinsic factors associated with predation efficiency, such as the development of radular teeth and venom potency, may also affect a predator’s ability to subdue certain types of prey [15, 17]. The significantly positive association between conotoxin gene expression and dietary diversity implies that prey diversity may exert pressure on conotoxin gene transcription. Intraspecific heterogeneity in venom composition has been suggested to represent local adaptation to different prey utilizing patterns among populations of snakes [3, 54]. But other studies contend that differences in venom composition among populations of snakes are unlikely to be driven by selection that derives from differences in predator–prey relationships, because few prey can escape envenomation and develop heritable resistance [55–57]. We postulate that high concentration and diversity of venom components within a single population may be beneficial to capturing a more diverse array of prey. Here we assume that mRNA quantities of conotoxin genes are positively correlated with the quantities of peptides, which holds true for venom genes of snakes [58–60] but has not been tested in cone snails.

Associations between venom composition and diet are not exclusive to cone snails, but in other venomous organisms the age-related shift in venom composition is coupled with shift in prey specialization rather than prey diversity. For example, increased quantities of neurotoxins in neonates/juveniles snakes may enhance the success rate of immobilizing small prey, while increased levels of pre-digestive components in adults may be more efficient for handling large endothermic prey [5, 51, 54, 61–63]. Changes in nematocyst ratios and venom compositions among differently sized individuals of the Australian jellyfish *Chironex fleckeri* and *Carukia barnesi* are coincident with prey shifts from invertebrate-based diets to a  vertebrate-based one [64, 65]. Unlike snakes and jellyfish, the strong coupling of conotoxin diversity and dietary diversity observed here has also been observed among species [20, 66] and among populations of the same species [27], and our study demonstrated that this mechanism is also applicable to individuals within a single population and through development.

Changes in conotoxin gene expression patterns exhibit a short delay relative to shifts in diet with growth, which suggests a potentially adaptive relationship between these two features. Environment-induced phenotypic and physiological variation often exhibits some delay relative to the environmental changes [67–70], and such a phenotypic change is advantageous when the delay is small [70]. The induced variation in phenotypes and physiology may result from transcription regulation [71–74], and the timing of gene regulation induced by environmental changes is different for genes affected. For example, increases in expression of heat-shock protein genes in yeasts occur almost immediately after heat exposure, but changes in expression of other genes occur some time after the exposure [72]. Therefore, conotoxin gene regulation may be facultatively induced by changes in dietary diversity. However, we cannot rule out the possibility that shifts in conotoxin gene expression represent a systematic process rather than one that is plastic with regards to changes in prey, nor the possibility that some of these conotoxin genes are used for defense rather than predation [18].

Moreover, the difference in timing of changes of conotoxin gene expression and diets is difficult to be quantified precisely. Frank [75] found that growth rates of *C. miliaris* exhibit a logarithmic relationship with shell lengths; during the first year of growth, shell length (up to 15 mm) is a linear approximation of growth rate. The lead of dietary shifts over changes of conotoxin gene expression reported here (the growth time of 1–2 mm in shell lengths) represents about 25 to 50 days of growth time if we assume growth rates of *C. ebraeus* and *C. miliaris* are similar. Nonetheless, increases in shell sizes of cone snail species can be abrupt and likely to be related to recent feeding bouts (personal communication by Alan Kohn). Therefore the difference in timing of dietary shifts and conotoxin gene regulation may be negligible.

Age-related variation of conotoxin gene expression and diet observed here can be confounded by several other factors. Because our sampling was performed at a specific time period, our data do not depict the seasonality in prey availability and prey choice. Moreover, we used a single gene, β-tubulin, as the endogenous control to quantify conotoxin gene expression under the assumption that expression levels of this gene are consistent among individuals [41]. If this assumption were not true, estimates of changes in conotoxin gene expression may be confounded by fluctuations in expression of the β-tubulin gene. Though levels of conotoxin gene expression are not significantly different among individuals that consumed different prey species, the limited number of individuals with known diets and conotoxin gene expression levels (i.e., *N* = 35) may reduce our power to detect an association, if any. In addition, age estimation of these individuals was solely based on shell lengths, variation of which within and among age classes may introduce noise in the real age-related patterns of changes in dietary specialization and venom gene expression. Experimental manipulation of predator–prey interactions and investigation of regulatory mechanisms of conotoxin genes may reveal more information about the evolution of venom gene expression in response to changes in prey specialization.

## Conclusion

In summary, *C. ebraeus* at Guam exhibited high variability in conotoxin gene expression against increasing shell sizes. The pattern of variation of conotoxin gene expression is largely associated with, and delays relative to, changes in dietary diversity with age. Though we cannot disentangle the systematic changes in development and selection pressure from prey capture, expression levels of conotoxin genes among individuals of a single population are positively correlated with dietary diversity rather than with specific prey species, a novel discovery worthy of further investigation.

### Online supplementary materials

Additional file 1: Tables S1-S3, Figures S1-S6 and Additional file 2 are available for download online.

### Availability of supporting data

16S rRNA sequences of fecal samples are deposited in GenBank with accession numbers KM364562-KM364584.

### Ethics

Collection of *C. ebraeus* samples has been conducted under the collection permits issued by the Department of Fishery and Wildlife Sciences at Guam, and the importation has been approved by the US Fish and Wildlife Service. All specimens have been deposited to the collections of the University of Michigan Museum of Zoology's Mollusk Division.

## Additional files

Additional file 1:
**Supporting figures and tables.** (DOCX 8211 kb) Additional file 2:
**Shell lengths of each sample and raw C values of the six conotoxin genes and the β-tubulin gene.** (XLS 37 kb)
