# Supplementary material for: Age-related association of venom gene expression and diet of predatory gastropods
Source: BMC Evol Biol. 2016 Jan 28;16:27. doi: 10.1186/s12862-016-0592-5 (PMC4730619; doi:10.1186/s12862-016-0592-5)
Supplement: Additional file 1: — Supporting figures and tables. (DOCX 8211 kb) [file 12862_2016_592_MOESM1_ESM.docx]

**Additional file 1**

**Age-related association of venom gene expression and diet of predatory gastropods**

Dan Chang, Thomas F. Duda, Jr

**Supplementary information:**

Supplementary Figures S1-S6.

Supplementary Tables S1-S3.

Supplementary Data File 1

**Supplementary Figures**

**Figure S1. Hierarchical clustering pattern of *C. ebraeus* individuals based on expression levels of six conotoxin genes relative to the β-tubulin gene.**

(A) Dendogram with the Wald’s method. The two major clusters are labeled at basal nodes.

(B) Histogram of shell lengths of individuals within cluster 1.

(C) Histogram of shell lengths of individuals within cluster 2


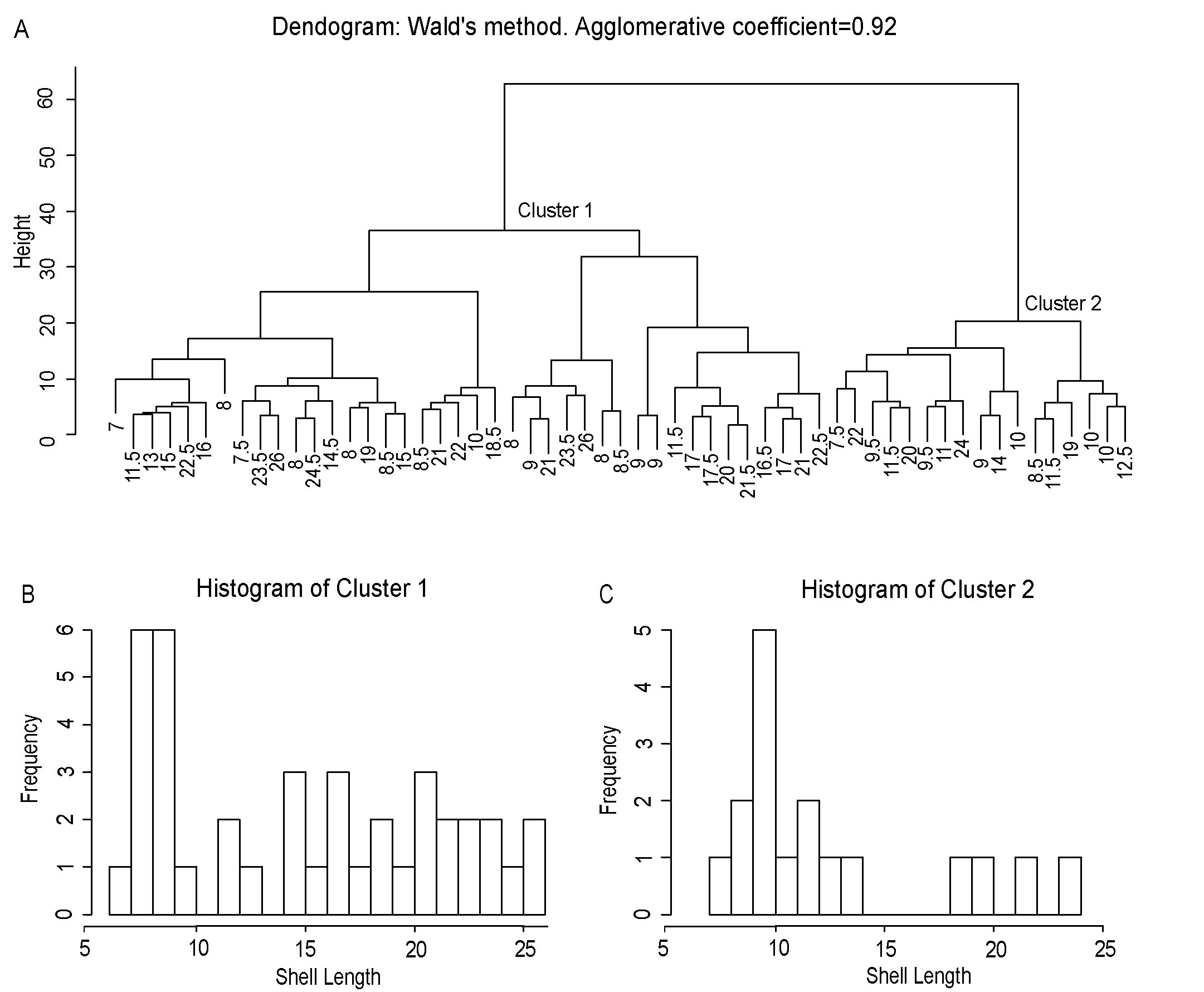


**Figure S2. Scatterplots and superimposed boxplots of expression levels of conotoxin loci of *C. ebraeus* individuals feeding on different prey species**. We superimposed a boxplot for each variable that has more than one sample.

**
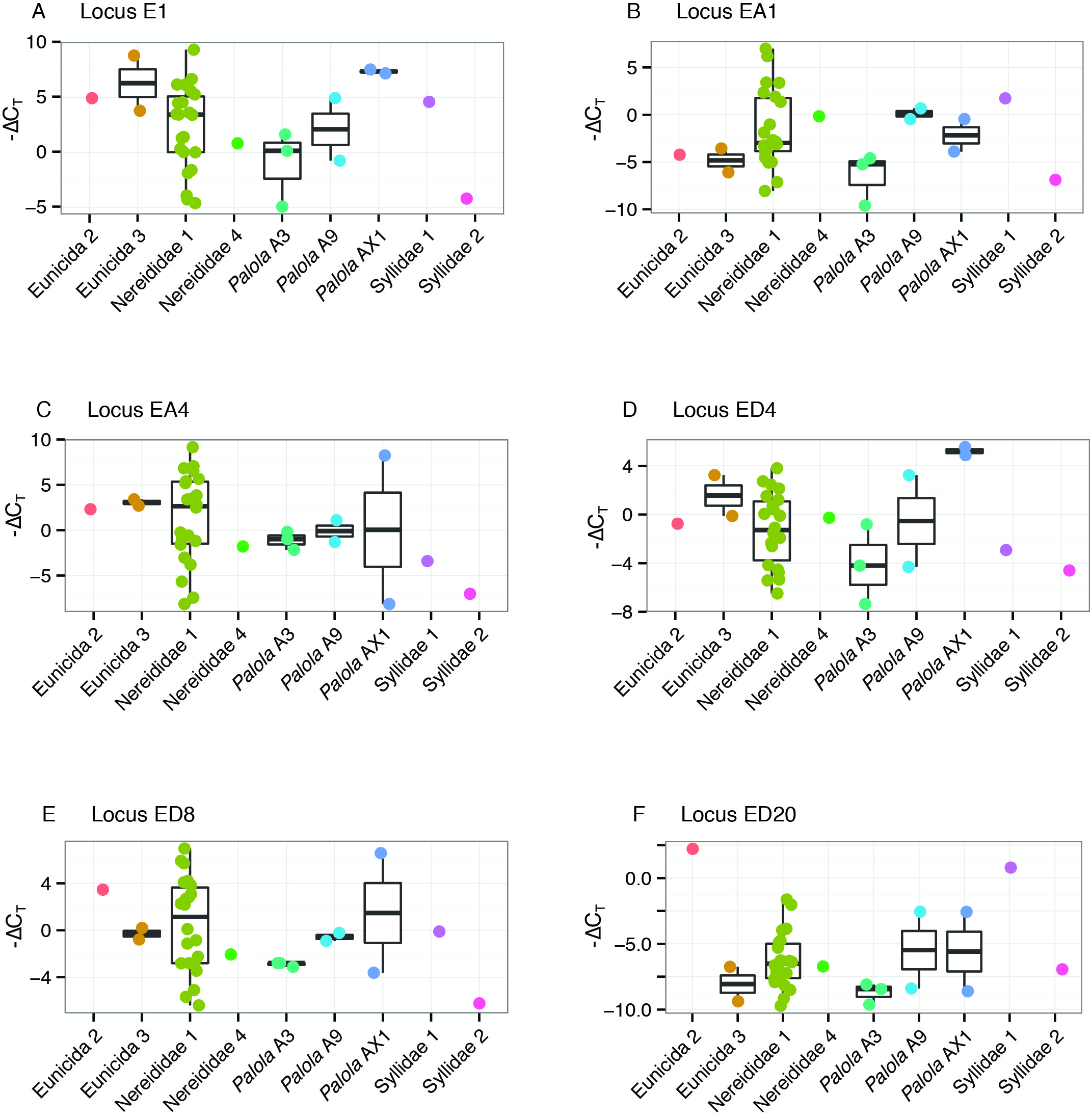
**

**Figure S3. Scatterplots and superimposed boxplots of relative expression levels of conotoxin genes E1, EA1 and EA4 among individuals feeding on different prey types.** Other Eunicida spp.: all Eunicida species except *Palola*. Eunicida: all Eunicida species. Non-Nereididae: prey species that are not of family Nereididae.

**
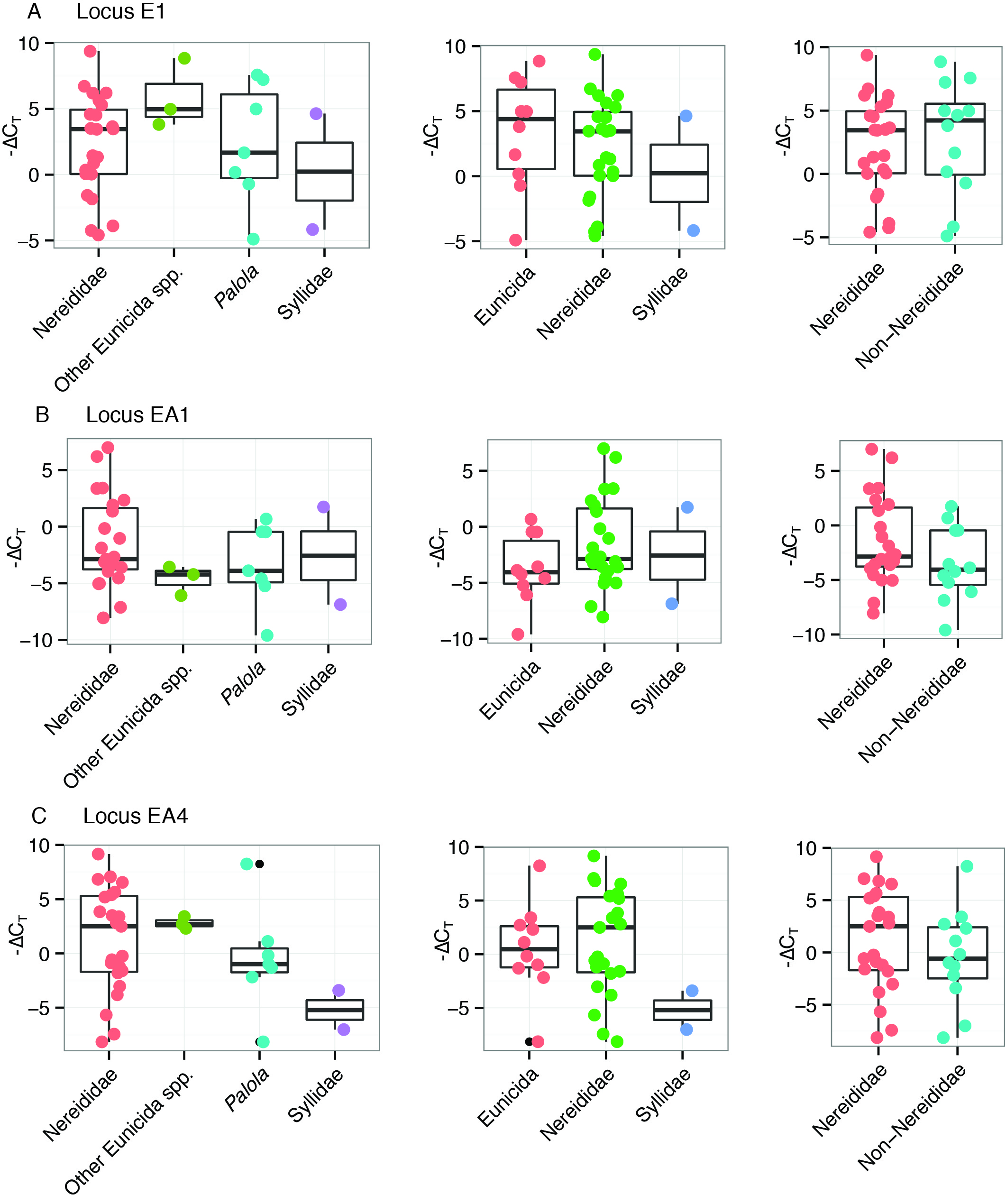
**

**Figure S4. Scatterplots and superimposed boxplots of relative expression levels of conotoxin genes ED4, ED8 and ED20 among individuals feeding on different prey types.** Prey type definitions are the same as in Figure S3.


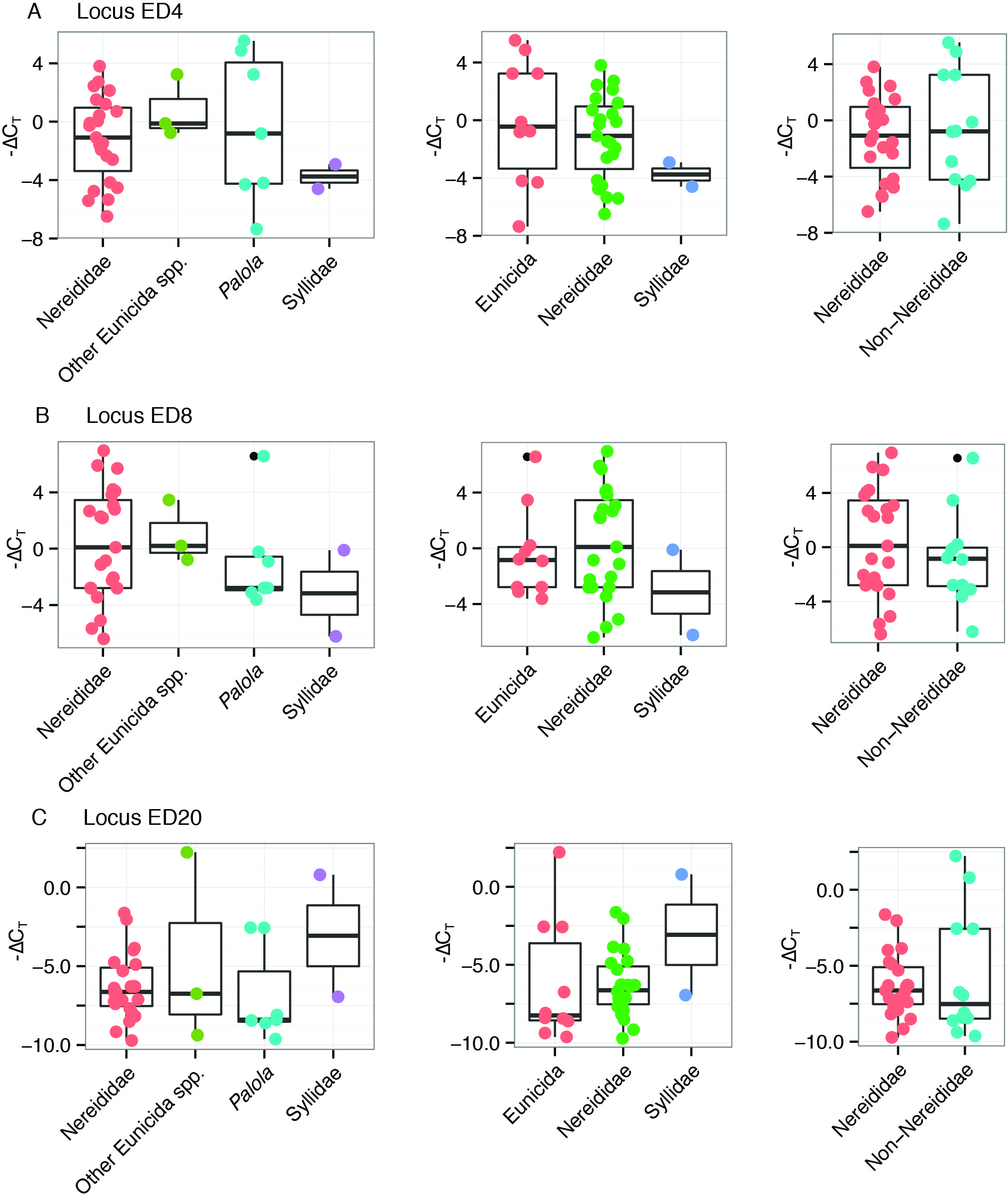


**Figure S5. Cross-correlation of the sliding window analysis of conotoxin gene expression levels and dietary diversities through increasing shell lengths.** Dietary variables include Shannon’s index (*H’*), Gini-Simpson’s index (*S*) and average genetic distances of 16S gene sequences of prey (GD). In each plot, Y-axis: correlation coefficient of two series; X-axis: lag in shell lengths of a dietary variable in comparison to conotoxin gene expression levels; blue dashed lines: 95% confidence intervals. Any vertical line exceeding the 95% confidence intervals represents a significant positive or negative correlation. Expression levels and dietary variables are centered and standardized.

**
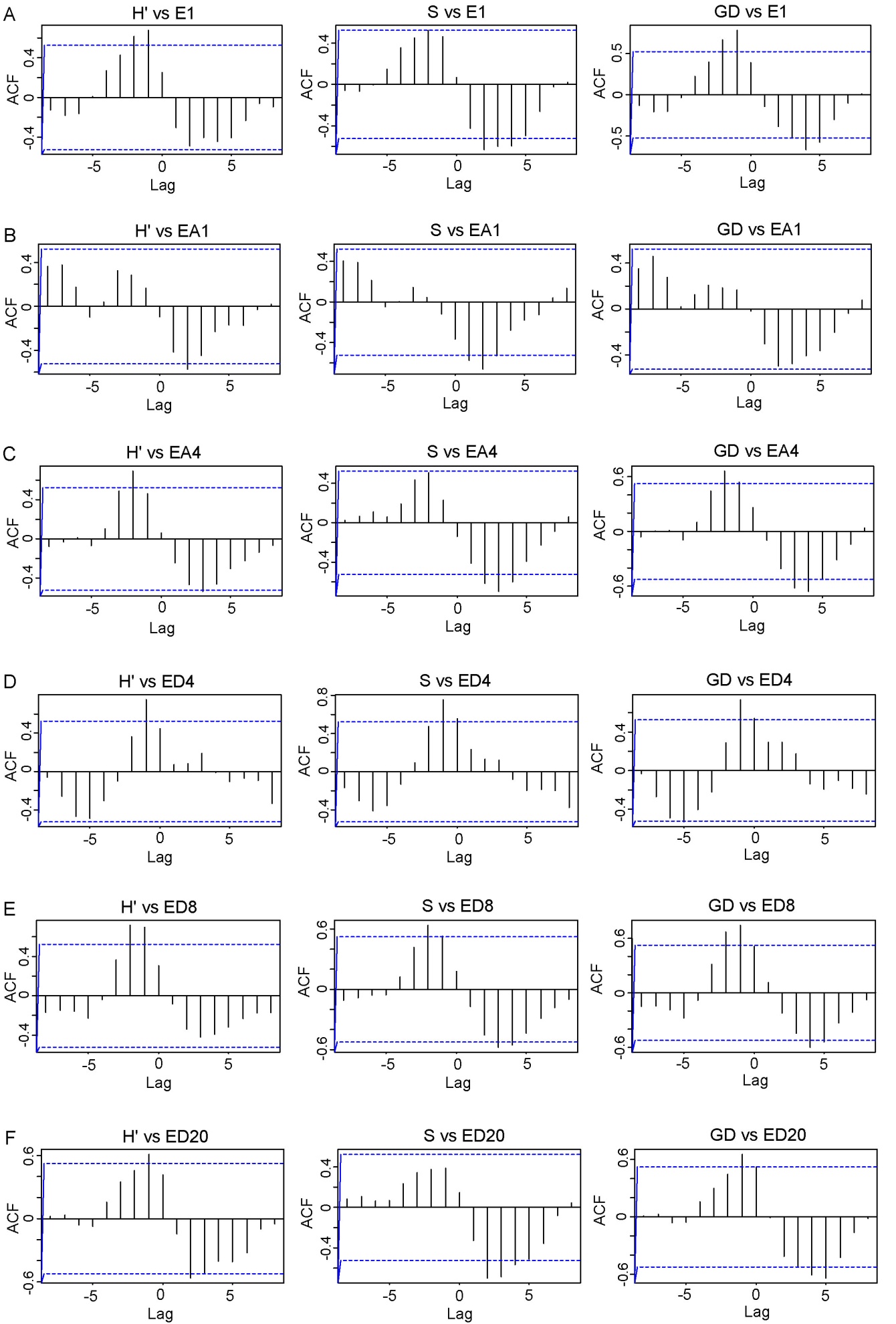
**

**Figure S6. Linear regression of specific lag of expression levels of each conotoxin locus with dietary variables (*S*, *H’* and GD).** The specific locus and lag are labeled on the Y-axis (e.g., ‘E1lag2’ in panel A means that changes in expression of the E1 gene fall behind the shifts of dietary diversity by a time period equivalent to 2 mm in shell lengths). Equations and R^2^ values of the linear regression are labeled next to the fitted trend lines. All slope variables are significant after correction for multiple testing except locus ED20 in (E). Expression levels and dietary variables are centered and standardized.

**
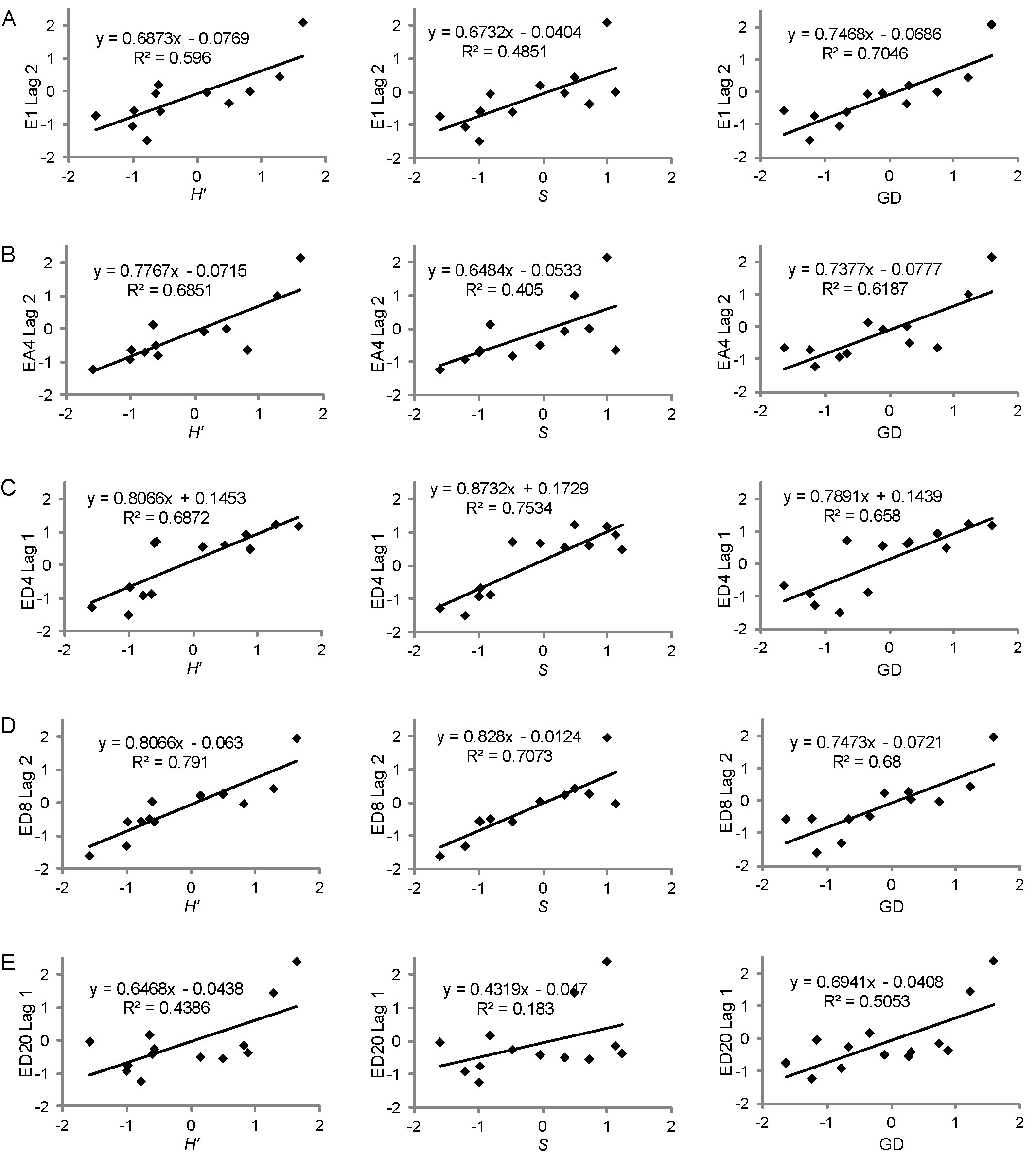
Supplementary Tables**

**Table S1. Primers used in qPCR to amplify the six conotoxin genes.** Putative conotoxin type encoded by each locus is labeled in parentheses in the ‘superfamily’ column. In the location column, ‘Toxin’ means the primer anneals to the toxin-coding region, while ‘Prepro’ refers to the prepro region of conotoxin genes. The IUPAC nomenclature code ([Johnson 2010](#_ENREF_1)) is used in primer sequences.

| **Type** | **Superfamily** | **Gene** | **Location** | **Primer Sequence (5’ to 3’)** |
| --- | --- | --- | --- | --- |
| Forward primer | O  (ω-conotoxin) | E1 | Prepro | CATCGTCAAGATGAAACTGACGTG |
|  | O  (δ-conotoxin) | ED4, ED8, ED20 | Prepro | CATCACCAAGATGAAACTGAC |
|  | A  (α-conotoxin) | EA1, EA4 | Prepro | ATGGGCATGCGGATGATGTTCAC |
| Reverse primer | O  (ω-conotoxin) | E1 | Toxin | ATCACGAAAGGGAAATATCAGGCG |
|  | O  (δ-conotoxin) | ED4 | Toxin | CATTACATAAGCCATTGCAGCATCC |
|  |  | ED8 | Toxin | CAACTAGAGGCAGACGTGGAAAAG |
|  |  | ED20 | Toxin | AGCTCAACTAGGCGCAGTTGAAAT |
|  | A  (α-conotoxin) | EA1 | Toxin | GGGTCCTGGAGCATCAGCCTTTA |
|  |  | EA4 | Toxin | TAKCAGCGTCTTCAACGACAATTC |

**Table S2. List of prey species identified from fecal materials with the DNA barcoding approach.** The unit of the shell length is millimeters. Prey species are defined in Fig. 1.

| **Shell Length** | **Prey**  **species** | **Shell Length** | **Prey**  **species** | **Shell Length** | **Prey**  **species** |
| --- | --- | --- | --- | --- | --- |
| 7.5 | Eunicida 2 | 11.5 | Nereididae 1 | 16.5 | Nereididae 1 |
| 9 | Eunicida 3 | 12 | Nereididae 1 | 16.5 | Nereididae 1 |
| 9 | Syllidae 2 | 12 | Eunicida 3 | 17 | Nereididae 4 |
| 9 | Syllidae 1 | 12.5 | Nereididae 1 | 17 | Nereididae 1 |
| 9 | Palola AX1 | 12.5 | Eunicida 1 | 18.5 | Palola AX1 |
| 9 | Eunicida 2 | 12.5 | Nereididae 1 | 18.5 | Nereididae 1 |
| 9 | Nereididae 1 | 13 | Nereididae 1 | 19 | Nereididae 1 |
| 9.5 | Nereididae 1 | 13 | Nereididae 1 | 19 | Palola A3 |
| 9.5 | Palola A9 | 14 | Nereididae 1 | 20 | Palola A3 |
| 10 | Nereididae 1 | 14 | Nereididae 1 | 21 | Nereididae 1 |
| 10 | Eunicida 3 | 14 | Nereididae 5 | 21 | Nereididae 1 |
| 10 | Nereididae 1 | 14.5 | Nereididae 1 | 21.5 | Nereididae 1 |
| 10 | Nereididae 1 | 15 | Nereididae 1 | 22 | Nereididae 1 |
| 10 | Nereididae 1 | 15 | Nereididae 1 | 22.5 | Palola AX1 |
| 11 | Nereididae 1 | 15 | Nereididae 1 | 22.5 | Palola A9 |
| 11.5 | Nereididae 1 | 15.5 | Nereididae 1 | 23 | Palola AX1 |
| 11.5 | Nereididae 1 | 16 | Palola AX1 | 24 | Palola AX1 |
| 11.5 | Nereididae 1 | 16 | Nereididae 1 | 24.5 | Palola A3 |

**Table S3. Results of the Fisher’s exact tests of prey compositions among size classes of *C. ebraeus* individuals.** Small: individuals with shell lengths less than 11mm. Medium: individuals with shell lengths between 11mm and 17mm. Large: individuals with shell lengths larger than 17mm. *P*-values were estimated by Monte Carlo simulations of 100,000 replicates in each test.

| **Null hypothesis** | **Alternative hypothesis** | ***P*-value** |
| --- | --- | --- |
| Small = Medium = Large | Small ≠ Medium ≠ Large | 0.001 |
| Small = Medium | Small ≠ Medium | 0.024 |
| Medium = Large | Medium ≠ Large | 0.004 |
| Small = Large | Small ≠ Large | 0.111 |

**Supplementary References**

Johnson AD (2010) An extended IUPAC nomenclature code for polymorphic nucleic acids. *Bioinformatics* **26**, 1386-1389.
